# Pyroptosis-related gene mediated modification patterns and immune cell infiltration landscapes in cutaneous melanoma to aid immunotherapy

**DOI:** 10.18632/aging.203687

**Published:** 2021-11-09

**Authors:** Jinzhi Meng, Xing Huang, Yue Qiu, Xifan Zheng, Junpu Huang, Zhenpei Wen, Jun Yao

**Affiliations:** 1 Bone and Joint Surgery, The First Affiliated Hospital of Guangxi Medical University, Nanning, People’s Republic of China

**Keywords:** cutaneous melanoma, pyroptosis, prognosis, immunotherapy, tumor microenvironment

## Abstract

Tumor occurrence, infiltration, and metastasis are significantly affected by the tumor microenvironment (TME). Increasing evidence has elucidated TME’s clinical significance in prognostic assessment and immunotherapy efficacy. Nonetheless, no studies have reported the potential pyroptosis-related genes (PRGs) function in TME immune cell infiltration. In this study, we systematically analyzed different PRG modification patterns in 685 cutaneous melanoma (CM) cases. We comprehensively explored the relationship between these PRG modification patterns and TME cell infiltration characteristics. Then, we used principal component analysis to construct a pyroptosis scoring system to quantify the PRG modification patterns in each CM patient. Three different PRG modification patterns were identified. Pyroptosis score was confirmed as an independent prognostic factor for CM patients. High pyroptosis score was characterized by high immunophenscore and more lymphocytes infiltration, such as T, B, and NK cells - indicating a strong ability to monitor and clear tumors, which may be responsible for the advantageous survival. Three independent cohorts that received immunotherapy confirmed the significant therapeutic efficacy and clinical benefit in high pyroptosis scores patients. This study revealed that the PRG modification patterns have a crucial effect on the CM complex and diverse microenvironment. Pyroptosis scores might serve as credible predictors of immunotherapy response and prognostic assessment. This provides a new direction for personalized immunotherapy strategies and appropriate immunotherapy candidates screening.

## INTRODUCTION

Cutaneous melanoma (CM) is the most aggressive skin cancer and accounts for 1.7% of the global malignancies incidence (approximately 232,100 cases). Worldwide, approximately 55,500 people die from malignant melanoma each year, accounting for 0.7% of cancer deaths [1, 2]. Recently, despite significant efforts to improve metastatic CM clinical outcome and prognosis, CM patients’ mortality rate has not improved and is rising [1, 3]. Immunotherapy can restore immune surveillance and kill cancer cells by the host’s natural immune system activation and tumor’s immune escape mechanism break [1, 4–6]. Also, it has become a breakthrough strategy in cancer treatment options, especially with the advent of anti-PD-1/PD-L1 targeting drugs, which are important to improve some CM patients’ overall survival. However, due to individual heterogeneity and tumor complexity, only a small proportion of patients can benefit from immunotherapy [6, 7]. Therefore, there is an urgent need for a reliable marker to identify suitable immunotherapy candidates to provide precise treatment.

Pyroptosis is a novel programmed cell death mode recently discovered [8, 9]. It is mainly characterized by non-selective pore channels formation in the cell membrane. This formation is mediated by the GSDMD pyroptosis driver, causing permeable cell swelling and rupture, and cell content release to further induce a powerful inflammatory response [8, 10]. Pyroptosis initiation can cause a major inflammatory factor (e.g. IL-1β and IL-18) release into the extracellular space, provoking an inflammatory response, and finally leading to diseases [11]. However, it can also mediate programmed cancer cell death to achieve tumor eradication by pyroptosis program inducing [12]. Additional evidence suggests that the pyroptotic cell death pathways key components, such as pro-inflammatory cytokines, gasdermins, and inflammatory vesicles, are also involved in cancer development and progression [13]. Inflammasome NLRP3, NLRP1, NLRC4, and AIM2 play a role in cancer pathogenesis by human immunity and programmed cell death regulation [14]. In CM, Inflammasome NLRP1 and NLRP3 variants are associated with progression risk, with the greatest correlation between NLRP1 and nodal CM. Inflammasome NLRP3 downregulation inhibits cancer cell development and hinders CM metastasis [15, 16]. Additionally, the pyroptosis pro-inflammatory effect was associated with TME regulation. The absence of GSDMD expression led to a decrease in the number of CD8+ T lymphocytes with reduced activity, as well as NK cells anti-tumor effects [17, 18]. Other studies have confirmed that the immune cell infiltration in TME is associated with tumorigenesis, metastasis, recurrence, and immunotherapeutic response [19]. For example, tumor-associated macrophages (TAMs) can secrete immunosuppressive factors to promote cancer progression, leading to poor prognosis [20, 21]. Conversely, T cells (e.g. CD8+ and CD4+), which infiltrated in tumor tissues, can recognize and clear abnormally developed cancer cells. They are associated with a survival advantage and are important in immunotherapeutic response [22]. However, immunotherapy resistance also occurs in patients with high TLS cells abundance, and TLS identification is not suitable for TME characterization in complex tumors [23, 24]. To date, PRGs mediated immune cell infiltration landscape in the CM microenvironment has not been elucidated.

A comprehensive analysis of the heterogeneity and complexity of the TME landscape under PRG modification patterns in CM would allow TME immune cell infiltration phenotype understanding, improving the ability to guide and predict immunotherapeutic responses. Additionally, these studies hold promise for reliable biomarkers discovery for appropriate screening of immunotherapy candidates and therapeutic targets. In our study, we integrated mRNA data from two cohorts, TCGA-SKCM and GSE65904, with 685 melanoma samples, synthesized PRG regulatory patterns, and explored the relationship between PRG modification patterns and TME immune cell infiltration phenotypes. We identified three different PRG modification patterns. TME cell infiltration differed significantly between the three patterns, corresponding to the immune inflamed (hot tumors), the immune excluded, and the immune desert (cold tumors) phenotypes. This suggested that PRG modification patterns can shape TME characteristics among different individuals. Therefore, we constructed a pyroptosis scoring system to quantify CM patients.

## RESULTS

### PRGs genetic variation landscape in CM

**Figure 1:**
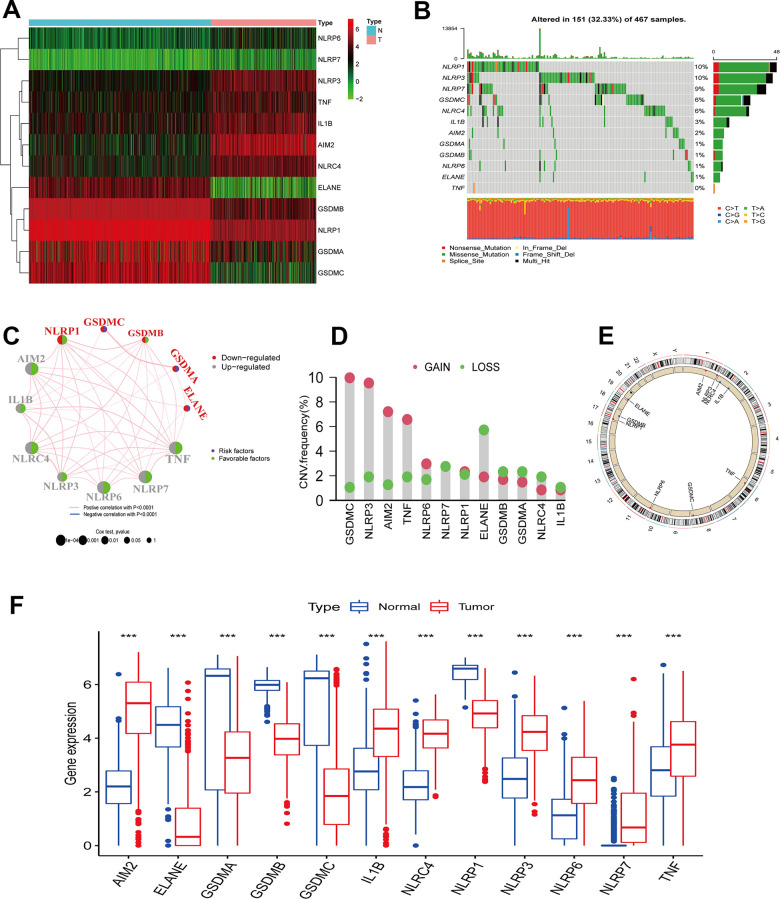
**PRGs genetic variation landscape in cutaneous melanoma.** (**A**) Heatmap of differential PRGs expression between normal skin tissue and cutaneous melanoma. Blue represents normal skin tissue, pink represents tumor tissue; upregulated genes were defined as red, and downregulated genes as green. (**B**) Mutation characteristics of 12 PRGs in the TCGA-CM cohort. The TMB is presented in the barplot at the top of the image; the mutation frequency of each PRGs is indicated on the barplot right. The barplot on the right represents different mutation types proportions. (**C**) Interaction circle diagram of the 12 PRGs in CM. Larger circles represent a greater CM prognostic impact. Dark blue inside the circle represents a risk prognostic factor and green a protective factor. The connecting lines between each PRG represent interactions. Pink represents the positive correlation between PRGs, and blue a negative correlation. Upregulated PRGs are marked in gray, and downregulated PRGs in red. p-values for Cox analysis ranged from: p < 1e-04, p < 0.001, p < 0.01, p < 0.05, p < 1. (**D**) PRG CNA variants frequency in the TCGA cutaneous melanoma cohort. Red: amplification frequency. Green: loss frequency. (**E**) CNA variants located on 23 chromosomes for 12 PRGs in the TCGA-CM cohort. (**F**) Expression of 12 PRGs between normal skin tissue and CM. Blue: normal skin tissue. Red: tumor tissue. (*p < 0.05; **p < 0.01; ***p < 0.001).

In this study, 12 differentially expressed PRGs - seven upregulated (AIM2, IL1B, NLRC4, NLRP3, NLRP6, NLRP7, TNF) and five downregulated PRGs (ELANE, GSDMA, GSDMB, GSDMC, NLRP1) - were screened for analysis (Figure 1A). We first investigated their CNV and mutations frequency in TCGA-CM. Among 467 TCGA-CM samples, 151 cases experienced PRG mutations, with a 32.33% mutation frequency. The top three highest mutation frequencies PRGs were: NLRP3 (10%), NLRP6 (10%), and NLRP7 (9%) (Figure 1B). Further analyzing PRGs prognostic role, we found that TNF and AIM2 were significantly associated with the CM patients’ overall prognosis. The PRG network diagram depicted an integrated landscape of PRG interactions and connections and their prognostic significance in CM patients (Figure 1C). From the CNV analysis results, CNV alteration was prevalent in the 12 PRGs, which showed mainly copy number amplification: AIM2, NLRP3, TNF, and GSDMC; while ELANE showed significant copy number reduction (Figure 1D). The 12 PRGs locations on the chromosome CNV changes are shown in Figure 1E. To understand if the PRGs expression was influenced by the genetic variants described above in CM patients, the PRGs levels between the samples from CM patients and normal skin tissues were determined. Our results showed that the alterations of CNV could be the prominent factors resulting in perturbations on the PRG regulators expression. (Figure 1F). Compared with normal skin tissue, CNV-amplified PRGs expression was significantly higher in CM (e.g. AIM2, NLRP3, NLRP6, TNF) and vice versa (e.g. ELANE, GSDMA, GSDMB) (Figure 1D, 1F). This analysis revealed significant PRG inheritance heterogeneity and variation between normal skin tissue and CM samples, suggesting that the expression imbalance of PRG played a crucial role in the occurrence and progression of CM.

### Construction of PRG molecular subclusters

**Figure 2:**
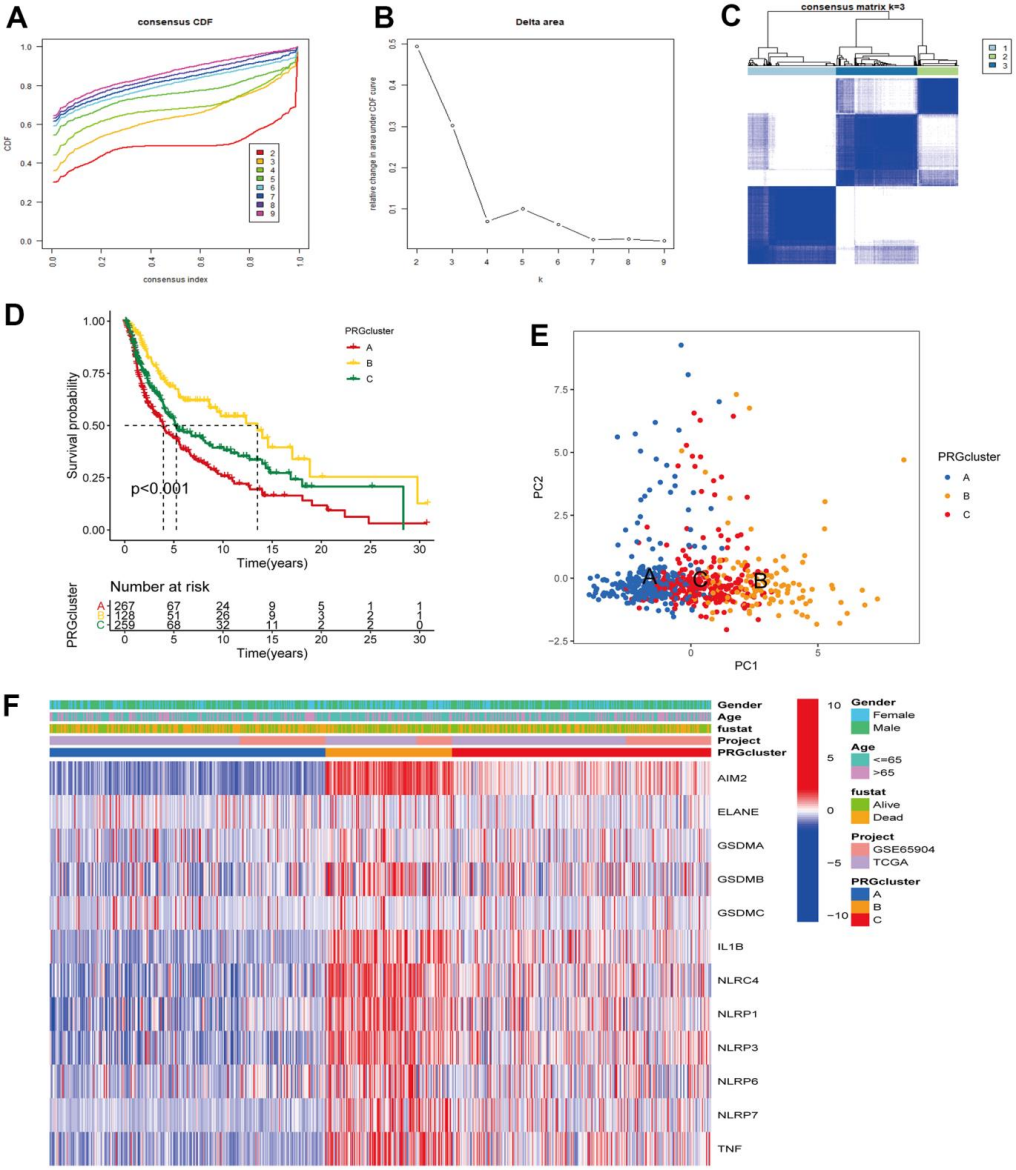
**Unsupervised cluster analysis classified CM patients into different subclusters.** (**A**, **B**) Consensus clustering cumulative distribution function (CDF) and relative change in the area under the CDF curve when K = 2-9. (**C**) Consensus clustering matrix in PRG modification patterns when K = 3. (**D**) Survival analysis curve of three PRG modification patterns in CM from TCGA, and GSE65904 cohorts. Red: PRG cluster A; orange: PRG cluster B; green: PRG cluster C. Log-rank p < 0.001, indicating a significant difference in OS between the three PRG modification patterns. Among them, cluster B OS was significantly better than A and C. (**E**) Principal component analysis (PCA) of three PRG modification patterns gene expression profiles. Blue represents PRG cluster A, orange PRG cluster B, and red PRG cluster C. (**F**) Unsupervised clustering heatmap of 12 PRGs in CM. PRG clusters, age, gender, and survival status were used as patient annotations. Red represents high PRG expression and blue low PRG expression.

**Figure 3:**
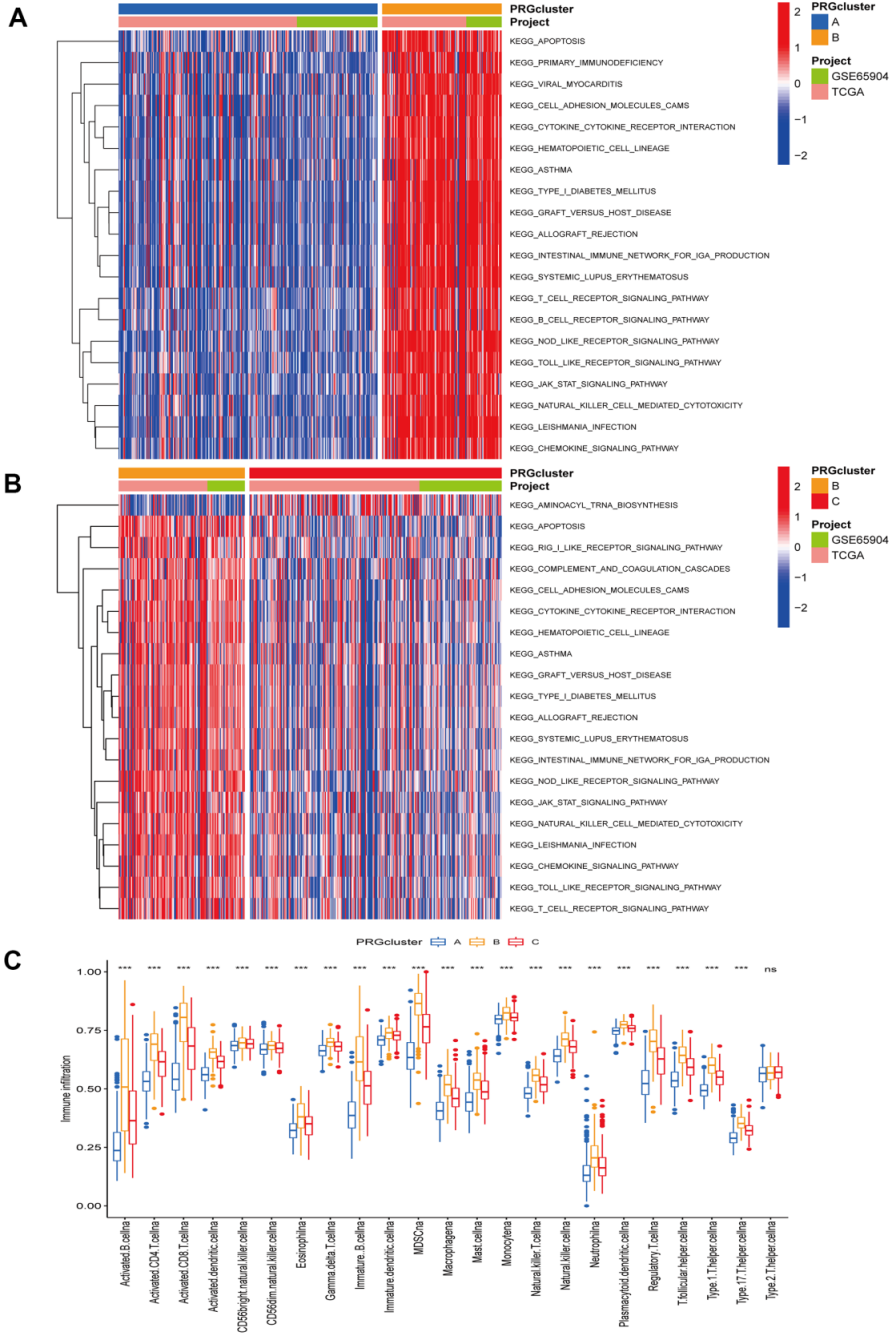
**Gene set variation and enrichment analysis.** (**A**, **B**) GSVA enrichment analysis annotated the biological functional pathways of different PRG modification patterns. Red represents functional pathways significant enrichment and blue represents small enrichment. (**A**) PRG cluster A vs PRG cluster B. (**B**) PRG cluster B vs PRG cluster C. (**C**) TME immune cell infiltration levels between the three PRG clusters. Blue represents PRG cluster A, orange PRG cluster B, and red PRG cluster C. The median value is represented as the thick line, and the interquartile range is represented as the box bottom and top. Scattered dots represent outliers. (*p < 0.05; **p < 0.01; ***p < 0.001).

The TCGA-CM data with complete OS information were integrated with the GSE65904 dataset into a meta-queue (n = 657). To classify CM patients into different subclusters, based on the 12 PRGs expressions in the meta-queue, we performed unsupervised cluster analysis using the “ConsensusClusterPlus” R package. Finally, CM patients were classified into three independent subclusters: PRG cluster A, PRG cluster B, and PRG cluster C; according to the principle of minimal crossover between cluster strata (Figure 2A–2C). Survival analysis between the three independent PRG clusters showed that cluster B had a significant survival advantage, followed by C, and finally A (Figure 2D). The transcriptome PCA between the three PRG modification patterns showed significant differences in transcription regarding different subclusters (Figure 2E). To further clarify the PRG modification patterns’ characteristics in different biological functions and clinical features, we provided systematic and comprehensive clinical annotations. These 12 PRGs were most highly expressed in cluster B, followed by C and, finally, A (Figure 2F). Subsequently, we applied GSVA enrichment analysis to explore the biological behavior among different PRG clusters. Significant PRG cluster B enrichment in pathways related to immune cell activation was observed, including, apoptosis, chemokine, natural killer cell-mediated cytotoxicity, T-cell receptor, and B-cell receptor-related signaling pathways (Figure 3A, 3B). Interestingly, the same cellular function pathways showed a diminished enrichment trend in clusters C and A (higher enrichment in C than A). Next, we used ssGSEA analysis to assess TME immune cell infiltration between different PRG clusters. Interestingly, a significant difference in immune cell content was observed between PRG subclusters. Moreover, almost all immune cells were significantly enriched in cluster B, followed by cluster C, and, finally, cluster A. Enriched cells included activated B, CD4, CD8 T cells, and immature B cells, similar to the GSVA enrichment result (Figure 3C). Furthermore, CM patients in this PRG cluster B modification pattern exhibited a matched prognostic advantage (Figure 2D). Previous studies have shown that tumor immunity is influenced by many factors, such as tumor type, host, and environment. In the cancer-immune cycle context, these factors are defined as immune phenotypes that can predict the tumor immunotherapy efficacy [25]. Altogether, cluster B was abnormally rich in innate immune cell infiltration - such as activated CD4, CD8, B cells, immature B cells - and matching favorable prognosis. Therefore, we hypothesized that the high immune cells content in PRG cluster B exerts an anti-cancer effect mediated by chemokines and exhibits a prognostic advantage - which corresponds to a tumor immune inflamed phenotype with the best immunotherapy response. Similarly, the phenotype associated with immune exclusion (e.g. presence of immune cells around the TME) was involved in PRG cluster C, but does not exert anti-tumor effects and responds poorly to immunotherapy. The immune desert phenotype was involved in PRG cluster A, including the lack of immune cells infiltration in TME, lack of antigen presentation, and unresponsiveness to immunotherapy.

### Identification of PRG phenotype-associated genes

**Figure 4:**
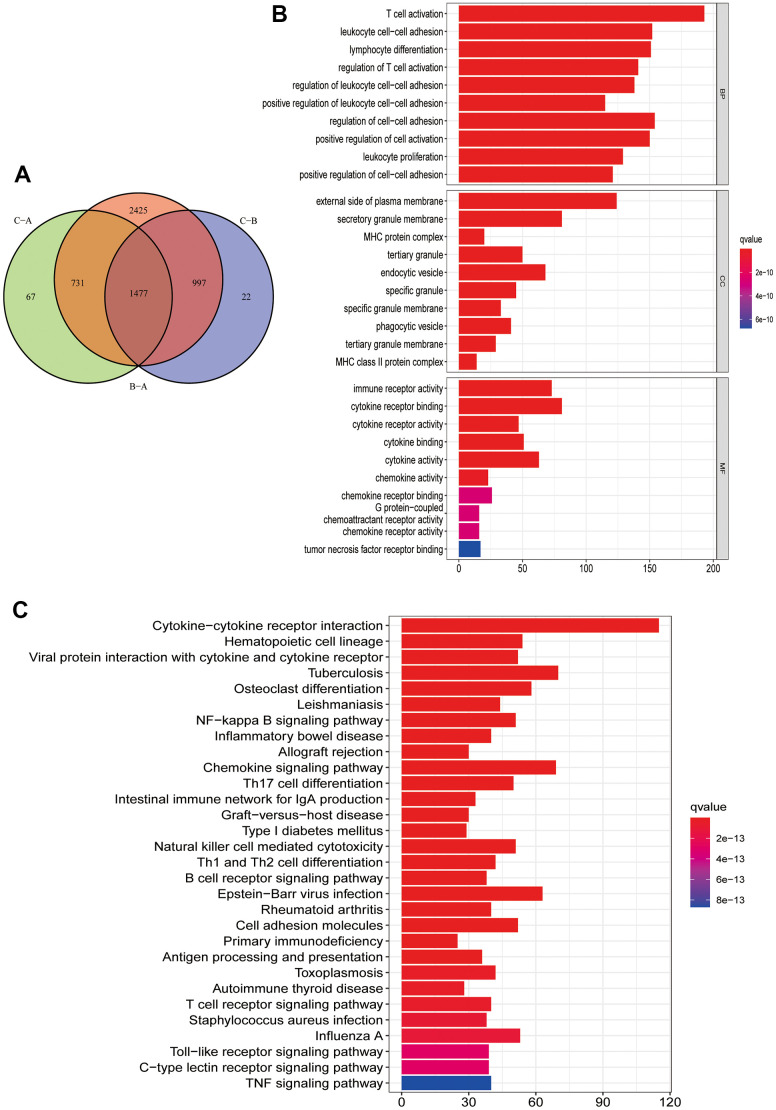
**Functional enrichment analysis and PRG signature genes annotation.** (**A**) Common intersection genes between the three PRG modification patterns. (**B**, **C**) PRG signature genes GO function and KEGG pathway enrichment analysis. The box color represents the number of enriched genes. Red represents a large number of genes enriched, blue is the opposite. (**B**) GO function enrichment analysis. (**C**) KEGG pathway enrichment analysis.

**Figure 5:**
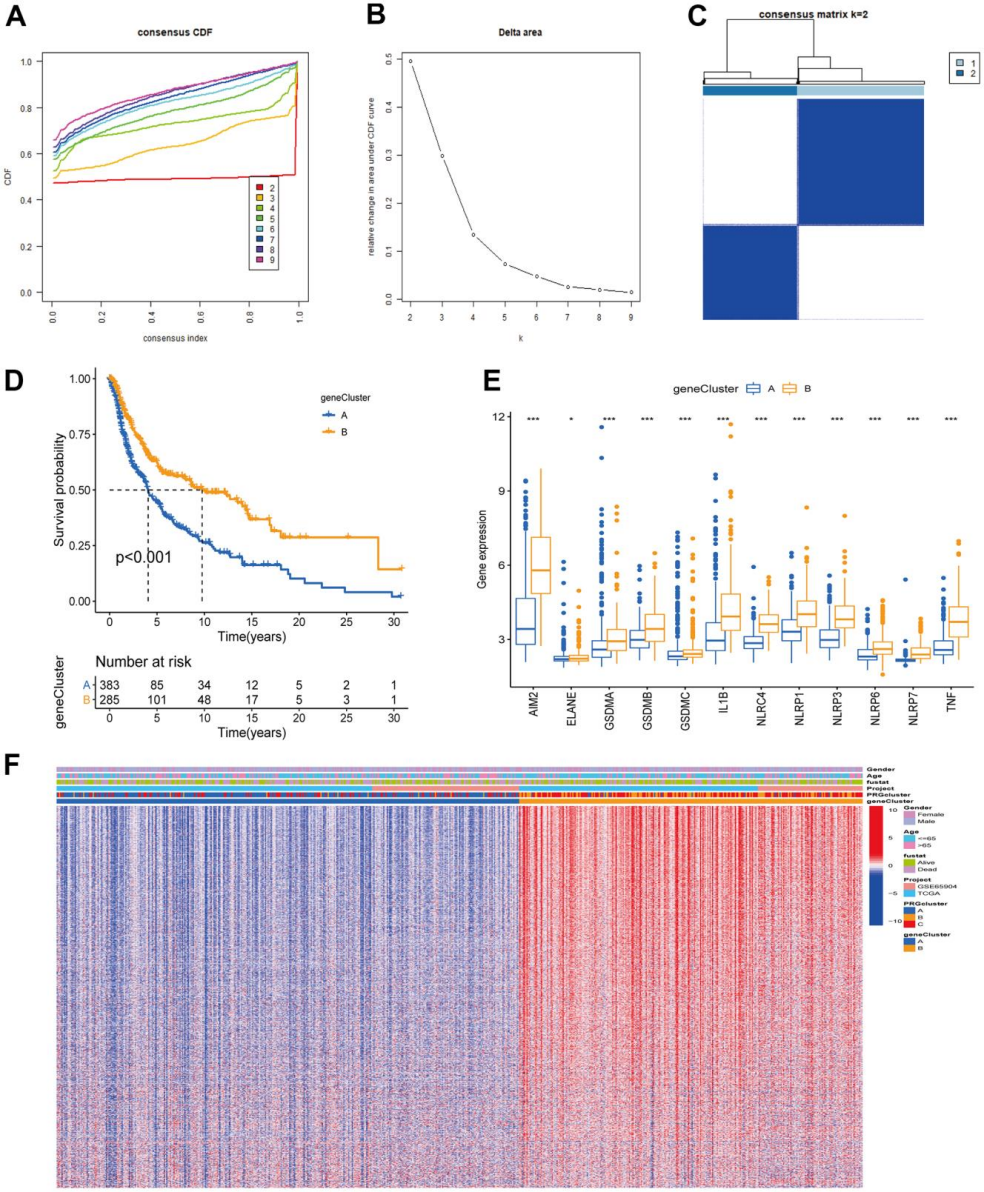
**Unsupervised clustering analysis based on PRG signature genes was used to classify CM patients into different gene clusters.** (**A**, **B**) Consensus clustering cumulative distribution function (CDF) and relative change in the area under the CDF curve when K = 2-9. (**C**) The consensus clustering matrix at K = 2. CM patients were divided into two gene clusters, defined as gene clusters A and B. (**D**) Survival analysis curves between gene cluster A and B. Blue: gene cluster A; orange: gene cluster B. Log-rank test p < 0.001 indicates that the OS between gene cluster A and B is significantly different. Gene cluster B OS was significantly better than gene cluster A. (**E**) Expression of 12 PRGs between gene clusters A and B. (**F**) Unsupervised clustering analysis heatmap of PRG signature genes. Age, gender, survival status, PRG clusters, and gene clusters are alternatively annotated. Red represents high gene expression, blue the opposite.

To further understand the potential biological functions among different PRG clusters, we screened 1477 PRG phenotype-associated DEGs using the “limma” R package, defined as PRG signature genes (Figure 4A). Then, to annotate and visualize DEGs’ biological functions, the GO functional and KEGG pathway enrichment analyses were performed using the “ClusterProfile” R package. Interestingly, significant enrichment was observed for DEGs in immune-associated processes. At the BP level, DEGs were enriched in biological processes involved in T cell activation and T cell activation regulation. At the CC level, DEGs were significantly enriched in MHC protein complexes associated with specific immune responses. In MF processes, DEGs were significantly enriched in immune receptor activity (Figure 4B). In KEGG pathway enrichment analyses, DEGs were also significantly enriched in pathways related to immune activity: natural killer cell cytotoxic activity, chemokine, B cell receptor, and T cell receptor-related signaling pathway (Figure 4C). Next, we screened prognosis-related DEGs using univariate Cox analysis and obtained 1257 prognosis-related DEGs. Based on the above 1257 prognosis-related DEGs, we performed an unsupervised cluster analysis to classify CM patients into two different gene clusters: gene cluster A, and gene cluster B (Figure 5A–5C). Survival analysis revealed that gene cluster B had superior OS than A (Figure 5D). All 12 PRGs showed overexpression in gene cluster B (Figure 5E). This suggested that high PRGs expression could be associated with superior CM patients’ prognoses. Moreover, the gene distribution heatmap revealed that these prognosis-related DEGs were highly expressed in gene cluster B (Figure 5F).

### Construction of PRG signatures scoring system (pyroptosis score)

**Figure 6:**
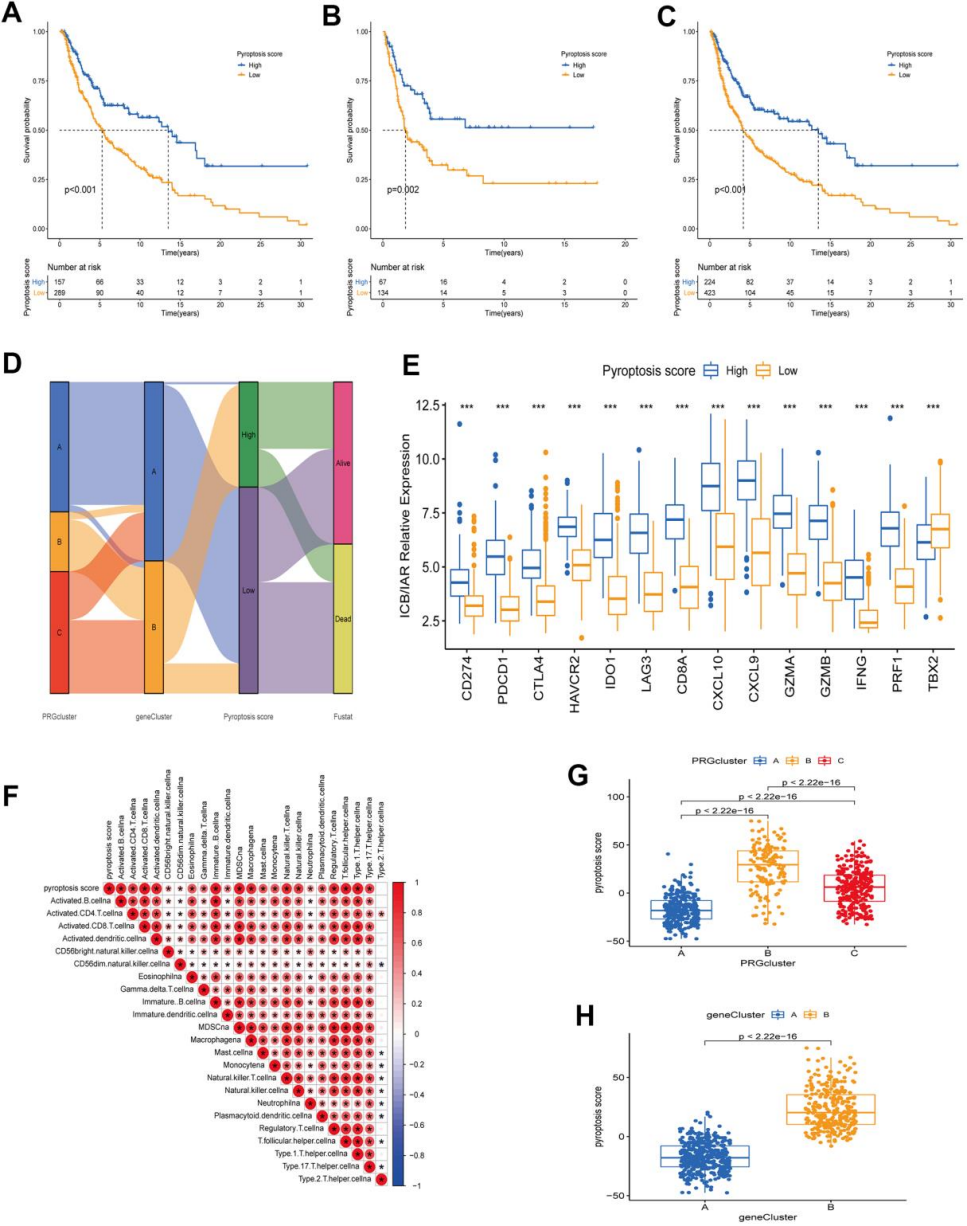
**Construction of a quantitative index pyroptosis score.** (**A**–**C**) Survival analysis curves of high and low pyroptosis score groups between different cohorts. Blue represents high scores, orange low scores. (**A**) TCGA cohort. (**B**) GSE65904 cohort. (**C**) TCGA combined with GSE65904 cohort. (**D**) Alluvial plots of the distribution of different PRG clusters, gene clusters, Pyroptosis scores, and survival outcomes. (**E**) Expression of immune check blocking genes (CD274, PDCD1, CTLA4, HAVCR2, IDO1, LAG3) and immune activation related genes (CD8A, CXCL10, CXCL9, GZMA, GZMB, IFNG, PRF1, TBX2) between high and low pyroptosis score groups. (**F**) Correlation analysis between TME immune cell infiltration and pyroptosis score. Larger circles in the box represent higher correlations. Red: positive correlation; blue: negative correlation. (**G**) Differences in pyroptosis scores between the three PRG modification patterns. The differences between the three subclusters were analyzed using the Kruskal-Wallis test (p < 0.001). (**H**) Differences in pyroptosis scores between two gene clusters (**A** and **B**). The differences between the two subclusters were analyzed using Wilcoxon rank-sum test (p < 0.001).

The PRG landscape quantitative indicators in CM patients were obtained by calculating two composite scores based on PRG phenotype genes using the PCA method: PC1 and PC2. The sum of PC1 and PC2 was defined as an independent score for each CM patient. Finally, we obtained a quantitative PRG landscape indicator for each CM patient, the pyroptosis score. Subsequently, according to the optimal score cutoff value, CM patients were divided into two groups: high and low pyroptosis score groups. Survival analysis in the TCGA-CM cohort showed that patients in the high-scoring group had better OS than those in the low-scoring group (Figure 6A). The same results were present in the GSE65904 cohort and the all-CM patients’ cohort (Figure 6B, 6C). Regression analysis indicated that pyroptosis score, age, T-stage, and N-stage were independent prognostic factors for OS in CM patients (Supplementary Table 2 and Supplementary Figure 1). The CM patients’ distribution in different modification patterns is shown in Figure 6D. Almost all PRG cluster B and gene cluster B patients were classified in the high pyroptosis score group, which was associated with a survival advantage. Further analysis revealed that pyroptosis scores differed significantly between PRG clusters and gene clusters. The highest median score was observed in PRG cluster B and the lowest in PRG cluster A (Figure 6G). From the above survival analysis, it could be seen that patients with high scores have a better overall prognosis (Figure 6A–6C). Therefore, we speculated that CM patients with PRG cluster B have a better prognosis and those with PRG cluster A have a worse one. This presumption is consistent with the results presented in Figure 2D. Similar speculations and conclusions were further validated (results shown in Figures 5D, 6H). Gene cluster B had a high pyroptosis score and a matched survival advantage (Figures 6H, 5D). After exploring the pyroptosis score prognostic value, we analyzed immune tolerance and activity in CM patients. First, we collected six immune checkpoint blockade (ICB) genes (CD274/PD-L1, CTLA4, PDCD1/PD-1, HAVCR2, IDO1, LAG3) and eight genes associated with the immune activation [Immune activation related (IAR): TBX2, PRF1, IFNG, GZMB, GZMA, CXCL9, CXCL10, CD8A] [23, 26]. We observed that all ICB genes were highly overexpressed in the high-scoring group. Also, ICA genes were overexpressed, except for TBX2, in the high-scoring group (Figure 6E). It is worth mentioning that ICB genes (CD274, CTLA4, PDCD1) are targets of anti-PD-1/PD-L1 and anti-CTLA4 antibody treatments. Therefore, we speculated that patients in the high pyroptosis score group are more suitable for immunotherapy. The correlation between pyroptosis score and TME immune cell infiltration was explored to provide preliminary validation. We found a positive correlation between pyroptosis score and the most innate immune cell infiltration levels, such as activated B, CD4 T, CD8 T, and dendritic cells, immature B cells (Figure 6F). High immune cell infiltration levels can provide a more potent anti-tumor effect, which may also explain why high pyroptosis score patients had a good prognosis.

### Correlations between pyroptosis score and different clinical features

**Figure 7:**
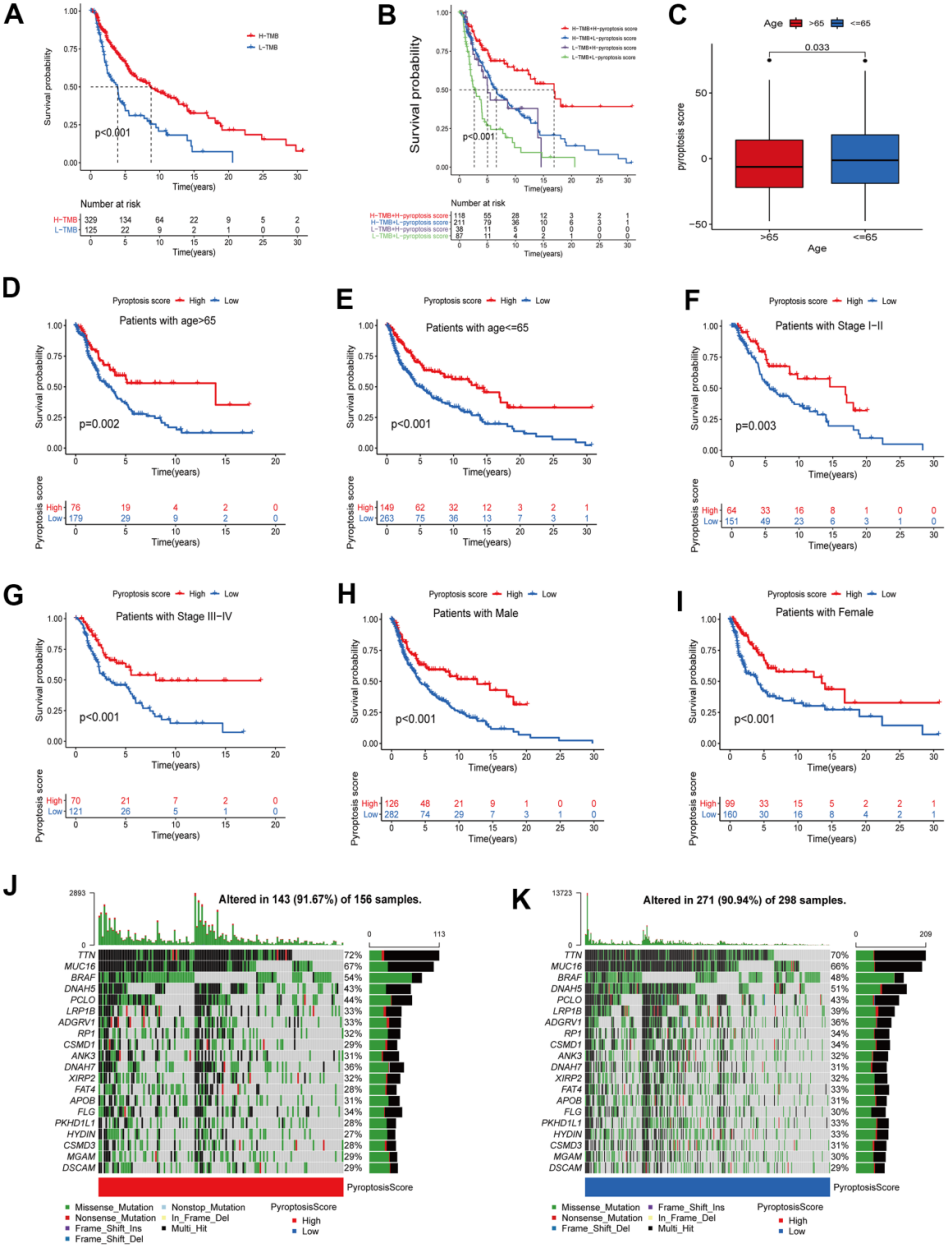
**Correlation analysis between pyroptosis score and different clinicopathological features.** (**A**) The survival between high and low TMB groups in the TCGA-CM cohort was analyzed using Kaplan-Meier curves (p < 0.001, Log-rank test). (**B**) Survival analysis of patients in the TCGA-CM cohort stratified by pyroptosis score and TMB was performed using Kaplan-Meier curves (p < 0.001, Log-rank test). (**C**) Differences in pyroptosis scores between age subgroups. The differences between the two subclusters were analyzed using Wilcoxon rank-sum test (p < 0.001). Red represents > 65 years and blue ≤ 65 years. (**D**–**I**) Survival curves between high pyroptosis score group and low pyroptosis score group for different clinical characteristics. The statistical difference was analyzed using a log-rank test. ((**D**) Patients with age > 65, p = 0.002; (**E**) Patients with age ≤ 65, p < 0.001; (**F**) Patients with stage I-II, p < 0.003; (**G**) Patients with stage III-IV, p < 0.001; (**H**) Male patient cohort, p < 0.001; (**I**) Female patient cohort, p < 0.001). (**J**, **K**) The waterfall plots of tumor somatic mutation were established based on the high (**J**) and low (**K**) pyroptosis score groups. The numbers on the graph represent the mutation frequencies. The box on the right represents the mutation types proportion. The barplot at the top shows TMB.

Evidence has demonstrated that tumors with high TMB are accompanied by correspondingly high CD8+ immune cell infiltration levels, thus acting as recognition and anti-cancer agent. This revealed that the TMB status is associated with the clinical response of patients receiving anti-PD-1/PD-L1 immunotherapy [27, 28]. High TMB alterations in tumors can enhance immunotherapy response and are associated with a long-term survival advantage and durable clinical benefits [6, 29]. Considering the TMB guiding significance in immunotherapy, we collected somatic mutation data of the TCGA-CM cohort to explore the relationship between CM-TMB and pyroptosis score. First, according to the optimal cutoff, patients in the TCGA-CM cohort were divided into a high and a low TMB group. Survival analysis showed that the OS between high and low TMB was significantly different, with patients in the high TMB cohort having longer OS than those in the low (Figure 7A). Next, we explored if there was a synergistic TMB and pyroptosis score effect in CM patients’ prognoses. Stratified survival analysis indicated that TMB levels did not affect the pyroptosis score independent predictive value. Significant differences existed between high and low TMB in pyroptosis score subclusters. Among patients with high pyroptosis scores, the OS was improved in high TMB patients compared to those with low TMB. Similarly, in the low pyroptosis scores group, high TMB patients had superior OS than low TMB ones (Figure 7B). Then, we analyzed the distribution of the somatic mutations in the TCGA-CM cohort between high and low pyroptosis scores using the “maftools” R package [30]. The top 20 driver genes with the highest mutation frequencies in the high and low pyroptosis score groups, and the corresponding mutation frequencies, are shown in Figure 7J, 7K. A broader TMB was observed in the high pyroptosis score group compared with the low one. These findings might provide new insights for immune checkpoint blockade therapy under pyroptosis phenotypic regulation mode.

Additionally, in the correlation analysis between pyroptosis scores and other clinical characteristics (age, stage, gender), patients with high scores showed a significant survival advantage. In the age group, we divided patients into > 65 years and ≤ 65 years groups. Pyroptosis scores were significantly different between age subgroups (Figure 7C). In the subsequent survival analysis, patients with high pyroptosis scores in both age subgroups showed better OS (Figure 7D, 7E). Similarly, we divided the patients into stage I-II and III-IV groups. The results of survival analysis revealed that patients in the high-scoring group had better prognostic survival (Figure 7F, 7G). The same results were obtained for the gender subgroups (Figure 7H, 7I). Collectively, the pyroptosis score not only predicts the long-term clinical patients’ outcome but more importantly, screens for suitable immunotherapy candidates.

### Pyroptosis score predictive value in tumor immunotherapy

**Figure 8:**
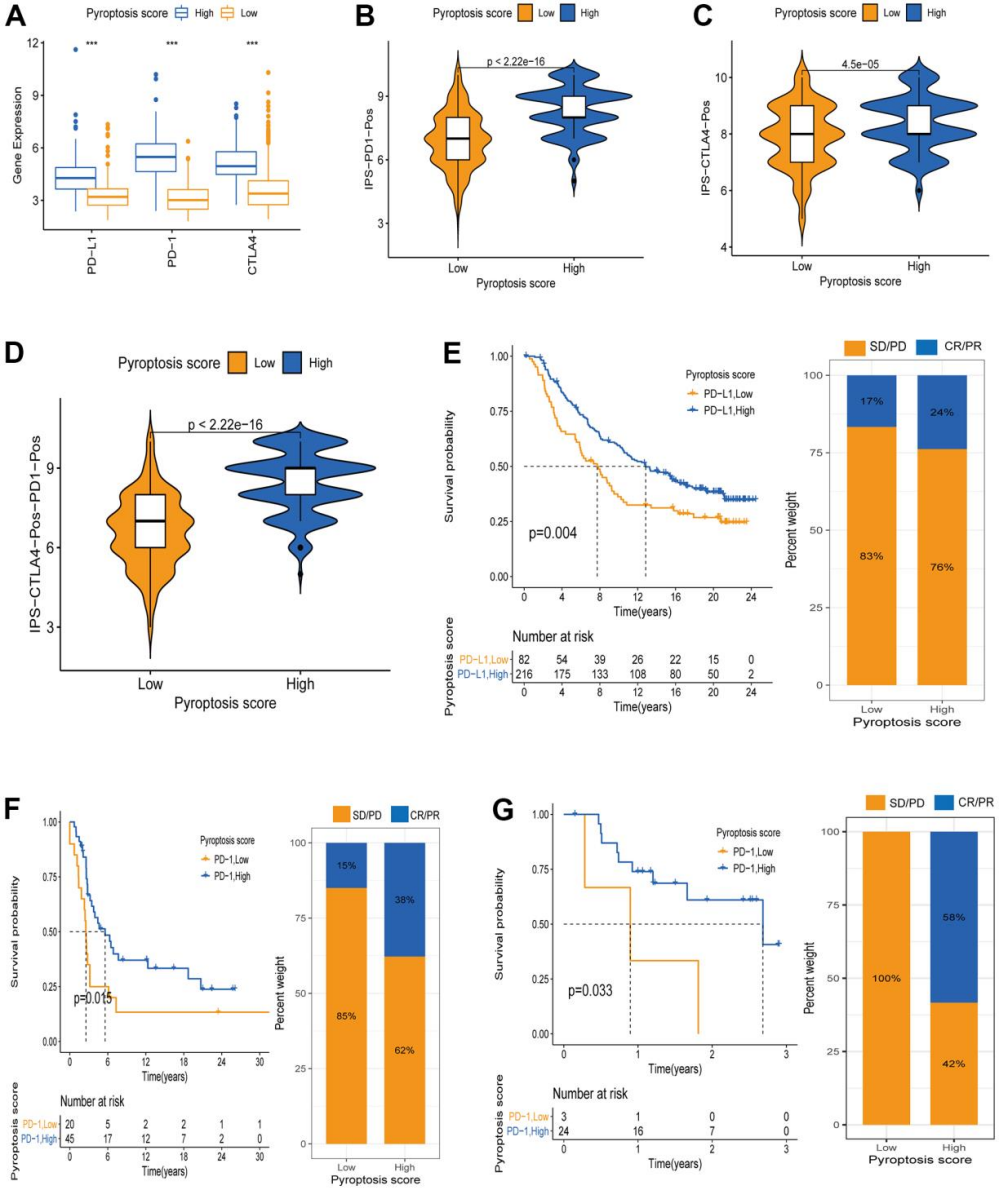
**Pyroptosis score predicts immunotherapy benefit in cancer patients.** (**A**) ICB genes expression (PD-L1, PD-1, CTLA4) between high and low pyroptosis score groups. (**B**–**D**) Immunophenscore (IPS) between high and low pyroptosis score groups. Blue represents the high-score group and orange the low-score group. The thick line within the violin plot represents the median value. The inner box between the top and bottom represents the interquartile range. (**B**) IPS score when PD-1 positive; (**C**) IPS score when CTLA4 positive; (**D**) IPS score when both PD-1 and CTLA4 positives. (**E**) Kaplan-Meier curves for survival analysis between high (n = 216) and low (n = 82) pyroptosis score groups in the IMvigor210 cohort receiving anti-PD-L1 therapy. Proportion of patients responding to immunotherapy between high and low pyroptosis score groups (SD/PD: stable disease/progressive disease; CR/PR: complete response /partial response. Response/Non-response: 17%/83% in low pyroptosis score; Response/Non-response: 24%/76% in high pyroptosis score). (**F**) Kaplan-Meier curves for survival analysis between high (n = 45) and low (n = 20) pyroptosis scores in the GSE93157 cohort receiving anti-PD-1 therapy (Response/Non-response: 15%/85% in low pyroptosis score; Response/Non-response: 38%/62% in high pyroptosis score). (**G**) Survival curves between the groups with high pyroptosis score (n = 24) and low (n = 3) pyroptosis score in the GSE78220 cohort receiving anti-PD-1 therapy (Response/Non-response: 0/100% in low pyroptosis score; Response/Non-response: 58%/42% in high pyroptosis score).

Recently, novel immunotherapies (represented by PD-1/PD-L1 blockade) have emerged clinically, showing unexpected results and improving advanced cancer patients’ disease and clinical outcomes. However, This therapy only partially response to a small fraction of patients [4, 6]. Considering the important value of novel immunotherapies in clinical treatment, we further explored the pyroptosis scores predictive efficacy on immunotherapy benefits. First, we explored the differential expression of ICB genes - CTLA4, PD-L1, and PD-1 - between patients with high and low pyroptosis scores using the Wilcoxon test. All three ICB genes showed overexpression in the high pyroptosis score group, indicating that those patients had a broader benefit when receiving anti-PD-1/L1 or anti-CTLA4 therapies (Figure 8A). Next, we used the CM-IPS cohort to assess patients’ immunophenscore (IPS) in different pyroptosis score groups. Results showed significant differences in IPS between the patients with high and low pyroptosis scores (Figure 8B–8D). CM patients treated with anti-PD-1 therapy and relatively high pyroptosis scores had higher IPS than those with low scores, indicating a superior therapeutic benefit in high-score patients (Figure 8B). Moreover, the same results were obtained when patients received anti-CTLA4 or anti-PD-1 therapy. This was another confirmation that the patients with relatively high pyroptosis scores were more suitable for immunotherapy (Figure 8C, 8D). Subsequently, we once more validated the pyroptosis score utility to infer the patients’ immunotherapy benefit. Patients receiving anti-PD-1/PD-L1 therapy in three separate cohorts (IMvigor210, GSE78220, and GSE93157) were divided into two groups based on their assigned pyroptosis scores. Interestingly, in the IMvigor210 cohort, better OS was observed in the patients with high pyroptosis scores after anti-PD-L1 therapy. Meanwhile, higher objective response rates (24%) were observed in the patients with high pyroptosis scores, compared to those with low scores (17%) (Figure 8E). The same results were observed for the GSE78220 and GSE93157 cohorts. Patients in the high pyroptosis score group had a better survival advantage and a higher objective response rate (Figure 8F, 8G). Altogether, our data strongly demonstrated that the pyroptosis score is a suitable biomarker for immunotherapy benefit prediction and significantly correlates with immunotherapy response.

## DISCUSSION

Increasing evidence shows that pyroptosis can induce tumor cells programmed death to inhibit cancer, and can also form a suitable TME around cancer to promote its growth. Regarding anti-cancer effects, pyroptosis has emerged as a potential tumor treatment strategy to improve advanced cancer patient’s clinical outcomes and prognoses [31, 32]. Since most studies on tumors are related to a single cell or a single regulatory factor, the overall TME infiltration and immunotherapy characteristics, under multiple pyroptosis regulation modes, are not completely understood. Comprehending the overall tumor biological characteristics and the immune cell infiltration level, under different pyroptosis regulation modes, would promote our immunotherapy efficacy understanding, and more effectively guide the clinical treatment plan selection.

In this study, we revealed three different pyroptosis modification patterns based on 12 PRGs expressions in CM. We also systematically analyzed the clinical significance and the TME immune cell infiltration level in different pyroptosis patterns. The immune cell content was significantly different among the three modification modes. PRG cluster B had high TME immune cell infiltration levels and was significantly enriched in immune cell activation pathways. The PRG cluster B characteristics corresponded to an immune inflamed phenotype (hot tumor) that responds well to immunotherapy. T cells are an important component of anti-inflammatory and tumor immunity and should enter the TME to effectively suppress tumor growth [33, 34]. Patients in the PRG cluster B had higher T-cell infiltration levels than those in clusters A and C, and thus have better resistance to tumors and show a matching survival advantage. Further, differential mRNAs in distinct PRG clusters are significantly associated with immune cell activation pathways and biological functions such as T cell activation and regulation. These differentially expressed genes were defined as PRG signature genes. Similar to PRG clustering analysis, two gene subclusters were identified based on the PRG signature genes. Their OS was also significantly different, with gene cluster B having a better prognosis. This demonstrated again that PRG regulatory patterns can consistently categorize patients and construct the TME. Therefore, a systematic and PRG regulatory pattern comprehensive analysis in CM can improve our understanding of the TME immune cell infiltration characteristics and guide personalized immunotherapy. Considering the individual TME differences under PRG modulation patterns, a quantitative metric is urgently required for each CM patient. Similar quantitative metrics have been established in breast and colorectal cancers to improve prognostic predictive efficacy [35, 36]. Thus, we constructed a scoring system based on PRG signature genes to assess PRG regulatory patterns in individual CM patients, the pyroptosis score. Multivariate Cox regression analysis revealed that the pyroptosis score was an independent prognostic predictor. PRG cluster B, characterized by immune inflamed phenotype, exhibited a high pyroptosis score. Furthermore, CM patients in the high pyroptosis score group and PRG cluster B showed a superior prognosis. IPS was positively correlated with immunogenicity, and its analysis revealed that patients in the high pyroptosis score group had a higher median IPS when receiving different immunotherapies, such as anti-PD-1, anti-CTLA4, or anti-CTLA4 combined with anti-PD-1. This suggested that patients with high pyroptosis scores have a better immune response and are more suitable for immunotherapy. The results above were validated in the IMvigor2210, GSE78220, and GSE93157 cohorts that received anti-PD-L1/PD-1 therapy [26, 37]. This suggested that our pyroptosis score is a very powerful and credible comprehensive assessment tool that might be used to determine the TME infiltration pattern in CM patients, bringing accurate predictions for CM immunotherapy response.

Our data also revealed that the pyroptosis score has a significant positive correlation with the T immune cell infiltration abundance in TME, such as activated CD4 T, CD8 T, and dendritic cells, natural killer T cells, regulatory T cells, and T follicular helper cells. Previous studies have shown that the main immune system strategy to monitor and eliminate tumors is: T cells recognize cancer antigens presented by dendritic cells, and activated T cells infiltrate into TME, causing peritumor chemokine production and interferon signaling, thereby disrupting the cancer cell population immune escape mechanism and mediating cancer cell death [38, 39]. Therefore, it is reasonable to believe that patients with high pyroptosis scores - characterized by a T-cell inflammatory phenotype - have a stronger ability to monitor and eliminate tumors. Also, targeted immunotherapy is more appropriate for this T-cell inflammatory phenotype. Additionally, the presence of the immunosuppressive factor PD-1/L1 may cause targeted CD8+ T-cell population dysfunction and cytotoxicity loss. PD-1/L1 targeted therapy can revitalize these dysfunctional CD8+T cells and restore their anti-tumor effect in TME [40, 41]. Moreover, there were significant differences in ICB genes (PD-1, PD-L1, CTLA4) expression between high and low pyroptosis scoring groups, with all three genes showing overexpression in the high pyroptosis scoring group. As previously reported, PD-1/L1 expression in cancer cells is an immune response biomarker to anti-PD-1/L1 therapy. Patients with positive PD-1/L1 had higher objective response rates and greater clinical benefit from anti-PD-1/L1 therapy [42, 43]. Based on these results, we suggested that CM patients with high pyroptosis scores, treated with anti-PD-1/L1, relieved the PD-1/L1 inhibitory effect on T cells populations, allowing them to resume their cytotoxicity and infiltrate the TME, mediating tumor cells pyroptosis or generating an inflammatory response. In recent years, a combination of radiotherapy (RT) and immunotherapy (IT) has been proposed for the treatment of CM. RT can increase the visibility of tumor antigens and enhance the immune response in CM [44]. However, there is no uniform standard for when to choose RT combined with IT. Some authors have classified the use of RT combined with IT into two main categories based on the time combination: peri-induction radiotherapy (PIR) and post-escape radiotherapy (PER) [45]. Our study can help to identify patients who are effective for IT and thus further improve the effectiveness of RT combined with IT therapy.

Briefly, the pyroptosis score can be used in clinical applications as an evaluation indicator for each patient’s PRG modification pattern and its corresponding immune cell infiltration characteristics to screen appropriate immunotherapy candidates and guide more effective treatment strategies. Additionally, we found that the pyroptosis score can be used to evaluate CM patients’ clinicopathological features, including age, gender, clinical stage, and tumor mutation burden. Similarly, the pyroptosis score can be used as an independent biomarker to assess CM patients’ prognoses. Exceptionally, we could also use the pyroptosis score to predict the treatment efficacy and clinical response of patients receiving anti-PD-1/L1 immunotherapy. In the future, our findings are expected to improve the CM patients’ response to immunotherapy and provide precise strategies for immunotherapy candidates.

## CONCLUSIONS

Overall, we comprehensively analyzed TME features in PRG modification patterns, demonstrated the PRG pattern regulatory mechanisms to shape different TME, and provided novel options for personalized immunotherapeutic strategies for CM patients. Additionally, the systematic analysis of PRG modification patterns in CM contributed to immune cell infiltration phenotypes understanding. Therefore, this study can help in the identification of suitable immunotherapy candidates and targeted therapeutic strategies through systematic evaluation of tumor PRG modification patterns, which could be important in clinical practice.

## MATERIALS AND METHODS

### Raw data acquisition and processing

The transcriptome RNA sequences (FPKM value) and the corresponding clinical data of 471 CM samples were retrieved from the TCGA platform. The GEO cohort (GSE65904) CM samples (n = 214) were obtained from the Gene Expression Omnibus (GEO, https://www.ncbi.nlm.nih.gov/geo/) platform. Additionally, data from 812 normal skin tissue samples were downloaded from the GTEx platform (https://xenabrowser.net/datapages/). The 33 original PRGs used in this study (Supplementary Table 1) were collected from previously published reviews [31, 32, 46, 47]. Samples with incomplete clinical information (Age, Gender, Stage, OS time) were removed, and the FPKM values in TCGA-CM were converted to Transcripts Per kilobase Million (TPM) values [48] and further used for copy number variation (CNV) analysis. After data correction, the transcriptome RNA sequences of the TCGA-CM and GSE65904 cohorts were merged. Raw data were processed using the R software version 4.0.2.

### PRGs unsupervised clustering analysis

Before clustering analysis, we screened differentially expressed PRGs (DEPRGs) in CM versus normal skin tissues. Due to the lack of normal skin tissue samples in the TCGA cohort, we downloaded 812 normal skin tissues from the GTEx platform. Data were uniformly transformed into TPM values before differential analysis. A total of 33 original PRGs were used, and, finally, 12 DEPRGs were screened for subsequent analysis under the criterion: |log2 fold change (FC) | ≥ 1 and false discovery rate (FDR) < 0.05. Next, we performed clustering analysis to construct CM patients’ PRG clusters based on the selected 12 PRGs expressions. The clustering algorithm in the “ConsensuClusterPlus” R package was used to determine subclusters number and stability. To ensure clustering accuracy, the analysis was repeated 1000 times [49].

### Gene set variation analysis (GSVA) and single-sample gene set enrichment analysis (ssGSEA)

GSVA analysis can be used to probe and annotate potential pathway functional enrichments between gene sets [50]. To understand the biological functions in different PRG clusters, we used the “GSVA” R package for each PRG cluster GSVA analysis. The signature genes enrichment in the “c2.cp.kegg.v7.3.symbols” gene set was analyzed using GSEA. Statistical significance was defined as *p* ≤ 0.05 (adjusted *p*-value). Additionally, the ssGSEA algorithm was used to evaluate the TME cells abundance between different PRG clusters. This algorithm allows the quantification of immune cell infiltration levels between gene sets [51]. In this case, we used “GSEABase” and “immune. gmt” R packages for analysis.

### Differentially expressed genes (DEGs) identification between different PRG clusters

We used the “limma” R package to screen DEGs between different PRG clusters *(p* ≤ 0.001). Then, biological functions were evaluated by Gene Ontology (GO) enrichment analysis, and regulatory pathways were analyzed by Kyoto Encyclopedia of Genes and Genomes (KEGG) enrichment analysis [52, 53].

### Pyroptosis gene signature establishment

To quantify pyroptosis gene modification patterns in each CM patient, a scoring system was established. First, DEGs were screened from different PRG clusters, and intersecting DEGs were retained for subsequent analysis. Univariate Cox regression methods were used to analyze the above intersecting DEGs and to screen for genes associated with CM prognosis. Subsequently, based on the prognosis-related genes, we used an unsupervised clustering approach to classify CM patients into different subclusters for a comprehensive systematic analysis. Furthermore, we applied Principal Component Analysis (PCA) to genes significantly associated with prognosis for pyroptosis relevant gene signature construction. The PCA method allowed to focus the scores on the highly correlated gene modules and to downscale modules with small contributions or low correlations. Principal Component 1 (PC1) and Principal Component 2 (PC2) were defined as positively and negatively correlated with pyroptosis gene signature, respectively. Finally, we defined pyroptosis scores for each CM patient using a method similar to the gene expression grade index [54]:


$$\text{Pyroptosis score}=\sum \text{PC}1_{\text{i}}+\sum \text{PC}2_{\text{j}}$$

Where i and j were defined as the genes involved in the pyroptosis phenotype.

### Pyroptosis score and other clinical features correlations

We retrieved the mutation data corresponding to TCGA-CM patients from the TCGA platform. The “maftool” [30] R package was used to analyze mutations between high or low pyroptosis scores patients. Results indicated the top 20 driver genes with the highest mutation frequency. Furthermore, we analyzed the OS between high and low TMB patients and TMB combined with pyroptosis scores. Meanwhile, the correlation between pyroptosis scores and other clinical characteristics (age, stage and gender) was also analyzed, including the proportion of patients with different ages and genders between high and low pyroptosis scores. Then, patients with different ages, stages and genders were classified into two groups (high and low scores) using the optimal cut-off value. Finally, the OS between them was analyzed. A *p* ≤ 0.05 was defined as significantly different.

### Collection of data sets and clinical data related to immunotherapy

The pyroptosis score predictive values were determined by analyzing four separate immunization-related datasets: IMvigor210 cohort [37], GSE78220 cohort [26], GSE93157 cohort, and CM Immunophenscore (CM-IPS). For the IMvigor210 cohort (n = 298), a dataset of advanced uroepithelial carcinoma treated with anti-PD-L1 antibody is available under the Creative Commons 3.0 License. Its corresponding expression data and full clinical information can be downloaded from http://research-pub.gene.com/IMvigor210CoreBiologies. The raw count data were normalized by the R package and further transformed into TPM values. For the GSE78220 cohort (n = 28), an RNA-transcriptomic dataset for metastatic melanoma anti-PD-1 anti-body treatment was obtained from the publicly available GEO platform (https://www.ncbi.nlm.nih.gov/geo/). Similarly, the GSE93157 cohort (n = 65), an immunotherapy cohort of patients with non-small cell lung cancer, head and neck squamous cell carcinoma, and melanoma receiving anti-PD-1 therapy, was downloaded from the GEO. The IPS were calculated on a scale of 0-10 based on representative gene expression from the immunophenogram, which provided a patient’s immune effect visual representation. CM-IPS data were retrieved from The Cancer Immunome Atlas (TCIA - https://tcia.at/home) public database.

### Statistical analysis

Comparisons between two different groups were performed using the Wilcoxon rank-sum test. For comparisons between three or more groups, the Kruskal-Wallis test was employed. The Kaplan-Meier method was used to perform Survival analyses related to pyroptosis scores. The survival statistic difference was analyzed using the log-rank test. The function “surv-cutpoint” was used to obtain the cohort’s optimal cut-off to classify patients into high and low pyroptosis score groups. This analysis was performed using the “survminer” R package. The prognostic value of pyroptosis score was evaluated using the Univariate and multivariate Cox regressions. The “forestplot” R package was used to visualize the results of univariate and multivariate Cox analyses. Mutation characteristics of 12 PRGs and PRG signature genes between high and low pyroptosis score subgroups were performed and visualized by the “maftools” R package. All data analyses were performed in the R software version 4.0.2. A *p*-value < 0.05 was defined as statistically significant.

## Supplementary Material

Supplementary Figure 1

Supplementary Tables
