# Supplementary material for: Pyroptosis-related gene mediated modification patterns and immune cell infiltration landscapes in cutaneous melanoma to aid immunotherapy
Source: Aging (Albany NY). 2021 Nov 9;13(21):24379–401. doi: 10.18632/aging.203687 (PMC8610130; doi:10.18632/aging.203687)
Supplement: Supplementary Figure 1 [file aging-13-203687-s001.pdf]

SUPPLEMENTARY FIGURE

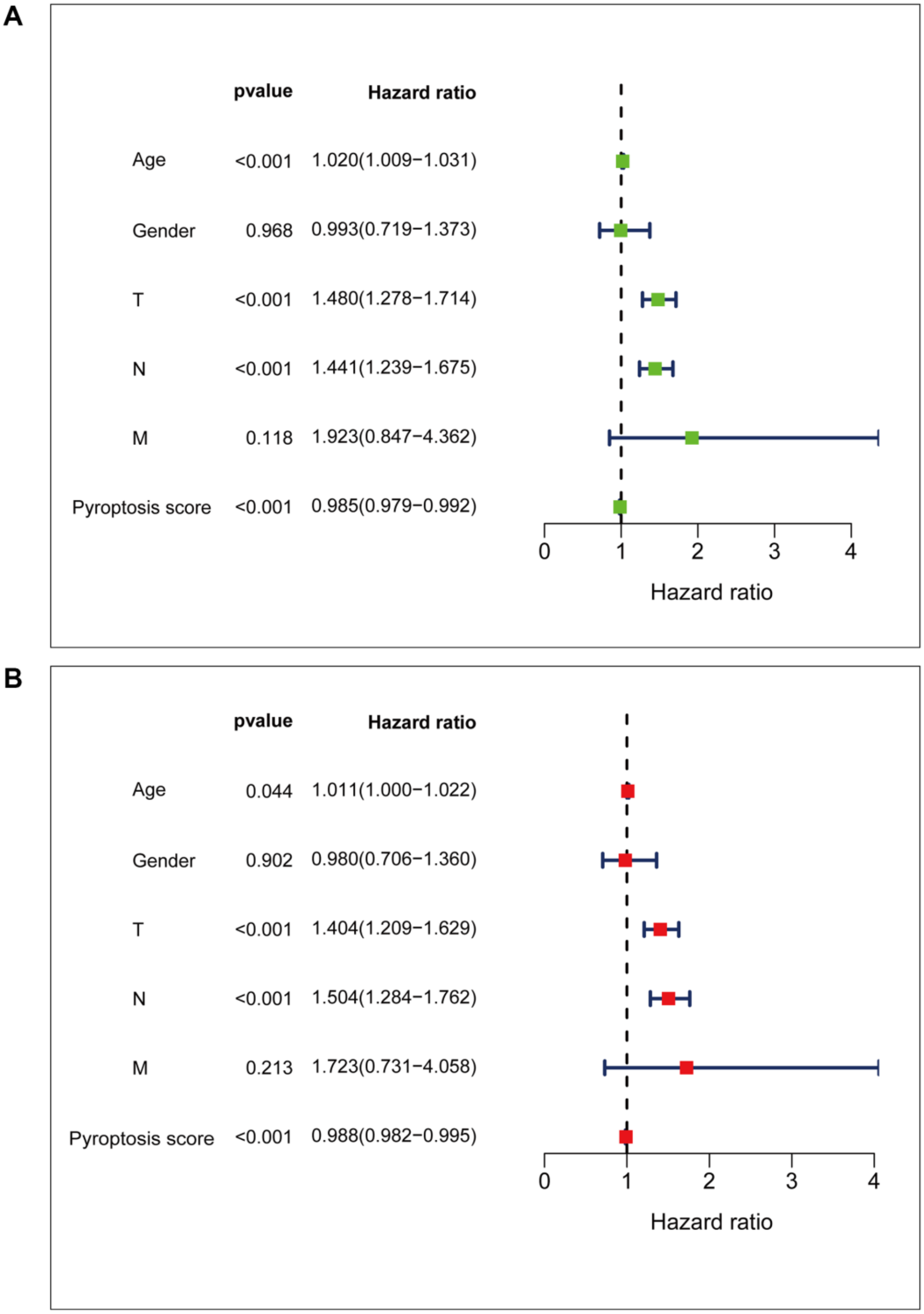

Supplementary Figure 1. Univariate versus multivariate Cox regression analysis to identify different clinical parameters prognostic value. (**A**) Univariate Cox regression analysis. (**B**) Multivariate Cox regression analysis.
