# Supplementary material for: Pyroptosis-related gene mediated modification patterns and immune cell infiltration landscapes in cutaneous melanoma to aid immunotherapy
Source: Aging (Albany NY). 2021 Nov 9;13(21):24379–401. doi: 10.18632/aging.203687 (PMC8610130; doi:10.18632/aging.203687)
Supplement: Supplementary Tables [file aging-13-203687-s002.pdf]

SUPPLEMENTARY TABLES

Supplementary Table 1. The 33 original pyroptosis-related genes that were used in this study.

Genes	Name
AIM2	Absent in melanoma 2
CASP1	cysteine-aspartic acid protease-1
CASP3	cysteine-aspartic acid protease-3
CASP4	cysteine-aspartic acid protease-4
CASP5	cysteine-aspartic acid protease-5
CASP6	cysteine-aspartic acid protease-6
CASP8	cysteine-aspartic acid protease-8
CASP9	cysteine-aspartic acid protease-9
ELANE	elastase, neutrophil expressed
GPX4	glutathione peroxidase 4
GSDMA	gasdermin A
GSDMB	gasdermin B
GSDMC	gasdermin C
GSDMD	gasdermin D
GSDME	gasdermin E
IL18	interleukin 18
IL1B	interleukin 1 beta
IL6	interleukin 6
NLRC4	NLR family CARD domain containing 4
NLRP1	NLR family pyrin domain containing 1
NLRP2	NLR family pyrin domain containing 2
NLRP3	NLR family pyrin domain containing 3
NLRP6	NLR family pyrin domain containing 6
NLRP7	NLR family pyrin domain containing 7
NOD1	nucleotide binding oligomerization domain containing 1
NOD2	nucleotide binding oligomerization domain containing 2
PJVK	pejvakin/deafness, autosomal recessive 59
PLCG1	phospholipase C gamma 1
PRKACA	protein kinase cAMP-activated catalytic subunit alpha
PYCARD	PYD and CARD domain containing
SCAF11	SR-related CTD associated factor 11
TIRAP	TIR domain containing adaptor protein
TNF	tumor necrosis factor

Supplementary Table 2. Univariate versus multivariate Cox regression analysis to identify different clinical parameters prognostic value.

Variables	Univariate analysis			Multivariate analysis		
	HR	95%CI	P-value	HR	95%CI	P-value
Age	1.099	1.009- 1.031	<0.001	1.011	1.000-1.022	0.044
Gender	0.993	0.719- 1.373	0.968	0.980	0.706-1.360	0.902
T	1.480	1.278-1.714	<0.001	1.404	1.209-1.629	<0.001
N	1.440	1.239-1.675	<0.001	1.504	1.284-1.762	<0.001
M	1.923	0.847- 4.362	<0.118	1.723	0.731-4.058	<0.213
Pyroptosis score	0.985	0.979- 0.992	<0.001	0.988	0.982-0.995	<0.001
